# Supplementary material for: Are carbon emissions trading and green financial instruments synergistic? -Comprehensive quantitative research based on content analysis
Source: PLoS One. 2024 Mar 7;19(3):e0298601. doi: 10.1371/journal.pone.0298601 (PMC10919601; doi:10.1371/journal.pone.0298601)
Supplement: S1 Data — (ZIP) [file pone.0298601.s001.zip › Data/地级市Z_F工作报告.zip!/║╙▒▒/╠╞╔╜╩╨/└┤╫╘╣1⁄2╓┌║┼í╛┬φ┐╦╩2╛▌═°í┐_2008.docx]

一、过去五年工作回顾

市十二届人大一次会议以来的五年，是唐山发展历史上不平凡的五年。在这五年里，三十一位党和国家领导人来唐视察工作，对唐山发展寄予厚望。在纪念抗震30周年之际，胡锦涛总书记亲临唐山，首次提出了建设科学发展示范区的战略构想，为唐山科学发展指明了方向。在这五年里，全市人民在市委的正确领导下，认真贯彻党中央、国务院和省委、省政府的一系列决策部署，抢抓机遇，开拓创新，拼搏进取，全面完成了市十二届人大一次会议确定的各项目标任务，经济总量跃居全省首位，曹妃甸大规模开发建设拉开序幕，科学发展示范区建设迈出坚实步伐，经济社会发展取得了令人瞩目的成就。

五年来，我们坚持狠抓发展第一要务，加快经济结构战略性调整和发展方式转变，综合经济实力跨上一个大台阶。全市地区生产总值由2002年的1102亿元跃升到2007年的2779亿元，年均增长14.6%;人均GDP由2002年的15715元增加到37734元;全部财政收入由83.2亿元增加到330.8亿元，年均增长31.8%，其中地方一般预算收入119.2亿元，是2002年的3.4倍。三次产业比例由2002年的17:51.7:31.3调整到10.3:57.4:32.3。农业农村经济全面发展。全面落实各项惠农政策，农业税、农业特产税全部取消。粮食产量稳步增长，2007年达到288.6万吨。乳业、瘦肉型猪、果菜、板栗、花生、水产品六大龙型经济健康发展，农业产业化经营率达到59%。畜牧水产业占农业总产值的比重达到49.7%，成为农业第一大主导产业。工业经济结构不断优化、质量效益显著提高。规模以上工业增加值达到1125.5亿元，实现利税551.3亿元，比2002年分别增长1.8倍和4.9倍。积极培育壮大产业链经济，冶金、建材、能源、装备制造、化工等主导产业竞争力明显增强。遵循国家产业政策，狠抓钢铁产业调整整合，淘汰了一批落后产能，行业整体素质大幅度提升，板带比由2002年的26.7%提高到60%。高新技术产业群体初具规模。服务业发展步伐加快。全社会消费品零售总额达到648.8亿元，年均增长15.7%。商贸流通、餐饮服务等传统服务业规模和水平不断提高，港口物流、金融、旅游等现代服务业发展步伐加快。我市被评为“中国优秀旅游城市”。项目建设支撑作用显著增强。五年间，全社会固定资产投资累计完成3214亿元，年均增长36.1%。其中，重点项目投资1054亿元，一批对市域经济发展具有较强支撑作用的重大项目相继建成，一批特色产业园区初具规模，形成新的经济增长群。节能减排取得积极进展。重点行业、企业节能环保技术推广收到明显成效，曹妃甸循环经济示范区建设和唐钢、开滦、冀东水泥等大型企业循环经济试点扎实推进。深入开展环保综合执法行动和治污减排安全整顿百日攻坚联合行动，对一批高污染、高耗能企业实施了关闭取缔、停产治理。2007年全市单位GDP能耗同比下降4.03%，二氧化硫、化学需氧量排放分别削减4.1%和4.9%。

五年来，我们深入实施生产力布局向沿海推进战略，以曹妃甸新区为重点的沿海开发取得重大突破，为唐山未来发展提供了战略支撑。曹妃甸开发建设列入国家“十一五”规划和首批循环经济试点园区，累计完成投资465亿元，进入大规模产业聚集的新阶段。唐山港总体规划、曹妃甸循环经济产业发展总体规划获国家批准。基础设施日趋完善，25万吨级矿石码头两个泊位建成运营，原油码头主体完工，煤炭码头建设进展顺利;吹填造地80平方公里，水、电、路、讯等各项基础设施基本配套。产业聚集实现突破，首钢京唐钢铁厂全面开工建设，二十二冶装备制造基地投产，德龙海洋工程等7个项目开工，荷兰EAM海洋装备、金维力油气钢管等20个项目签约。滨海新城选址、规划等前期工作取得重要进展。统筹沿海一线开发，谋划实施曹妃甸新区发展战略。在曹妃甸“龙头”带动下，沿海一线开发建设全面提速，唐山港吞吐能力攀上亿吨大港台阶，整个沿海地区强势崛起的态势正在形成。

五年来，我们全面深化各项改革，努力扩大双向开放，经济发展活力进一步增强。各项改革取得新成效。在全面完成1154家市县(区)属国有企业改制的基础上，以产权制度改革为核心，对188家企业进行二次改制，对31家困难企业实施改制攻坚。下岗职工基本生活保障与失业保险顺利并轨。国有资产监管体系进一步健全。行政管理体制改革取得明显成效，事业单位改革稳步推进。要素市场健康发展，流通、财税、金融、投融资等各项改革不断深化。民营经济快速发展，增加值占全市生产总值比重达到67%，比2002年提高29个百分点。双向开放打开新局面。五年间，实际利用外资22.9亿美元，批准三资企业项目267个，是对外开放以来利用外资最多的时期。对外开放广度深度不断拓展，在新加坡、日本、韩国以及港澳等地区组织多形式的招商活动，成功举办了第六至第十届唐山中国陶瓷博览会和河北·唐山(曹妃甸)经贸洽谈会，与一批国内外知名大公司大集团等战略投资者成功对接，爱信汽车零部件等42个投资千万美元以上项目相继落户唐山。区域经济合作取得重要成果，与天津滨海新区、秦皇岛、承德、廊坊等地区以及国家发展银行、中材集团等企业签订了战略合作框架协议。外经工作领域和规模进一步扩大。对外贸易快速发展，全市进出口总额达到51.9亿美元，其中出口28.2亿美元，分别比2002年增长6.1倍和6.3倍。

五年来，我们围绕建设适宜人居和创业的现代化滨海城市，加快推进城镇化进程，城乡面貌显著改观。全市城镇化率达到49.5%，比2002年提高8.5个百分点。新一轮城市总体规划修编取得阶段性成果，确立了以市区和曹妃甸生态城为双核，北部山前城市带、南部临海城市带协调发展的“双核两带”城市空间新布局。五年间，累计投资500多亿元，相继完成了城乡路网、城市供水、污水处理、集中供热、燃气输配和城市绿化美化亮化等一批重点工程，城市功能不断完善。我市被命名为国家园林城市，南部采沉区生态建设项目荣获联合国“迪拜国际改善人居环境最佳范例奖”。特别是去年组织实施了以震后危旧平房改造和旧小区集中供热、拆违拆迁、城市出入口和建设路景观大道改造等为重点的城市建设改造攻坚战，提升了城市人气和城市形象。县城和小城镇建设水平明显提高。新建改建农村公路6115公里，在全省首批实现村村通公路和村村通客运班车。扎实推进文明生态村建设，累计投入28.5亿元，开展创建的村达到3033个，占行政村总数的53%，农村生产生活环境明显改善。

五年来，我们统筹经济社会协调发展，大力加强各项社会事业建设，促进了社会和谐进步。科技创新能力不断增强。五年全市财政用于科技的投入达到9.8亿元，比前五年增长1.1倍，累计取得737项关键技术创新成果，获省级以上科学技术奖134项。教育事业全面发展。2007年全市财政用于教育支出35.9亿元，是2002年的3倍。九年义务教育水平得到巩固提高，农村免费义务教育全面实现，初中毕业生升学率达到92.6%，基本普及高中阶段教育，河北理工大学等5所高等院校通过教育部办学水平评估。人才培养、引进取得新成效，人才队伍不断壮大。医疗卫生事业全面发展。全市财政累计用于卫生支出39.4亿元，比前五年增长1.8倍，重点加强公共卫生、医疗服务体系建设，城乡医疗卫生水平进一步提高。抗击“非典”取得重大胜利。文化事业繁荣发展。《人影》、《香妃与乾隆》等一批优秀作品获国家级大奖。群众文化活动蓬勃开展，成功举办了第二至第五届中国评剧艺术节、2005中国国际皮影艺术展演和社区文化艺术节。广泛开展精神文明创建活动，全社会文明水平不断提高。成功承办了第十二届省运会，获得体育成绩与精神文明双丰收。全市计划生育率保持在96%以上，人口自然增长率年均5‰左右。广播电视、民族宗教、外事侨务、爱国卫生、防震减灾、妇女儿童、老龄、气象、档案、人防、民兵预备役和国防动员等各项工作都取得了新的成绩。

五年来，我们坚持以人为本，高度重视解决民生问题，人民群众幸福指数不断提高。城镇居民可支配收入和农民人均纯收入分别达到14235元和5825元，年均分别增长13.8%和9.9%，是近年来增长最快的时期。家庭财产普遍增加，城乡居民消费结构升级加快，住房条件进一步改善，汽车、电脑等现代消费品进入普通百姓家庭。就业和再就业工作收到实效，城镇累计新增就业27万人、安置下岗失业人员再就业15万人，城镇登记失业率控制在4.5%以内，零就业家庭就业问题基本得到解决。着眼于改善民生，坚持每年组织实施实事工程，五年共为群众办实事125件，重点解决了一批事关群众切身利益的教育、医疗卫生、社会保障等方面的实际问题。累计投入6.5亿元，改造农村中小学危房99.2万平方米，在全省率先全部消灭农村中小学危房;投入2.1亿元，对135所乡镇卫生院实施了标准化改造，在全省率先实现乡镇卫生院达标;投入3.5亿元,对101所农村敬老院进行了整合改造，在全省率先实现农村五保户全部财政供养;全面推行农村新型合作医疗制度，农民参合率达到93%，启动实施了城镇居民医疗保险制度，在全省率先实现城乡居民医疗保险制度全覆盖;先后7次提高城镇居民最低生活保障标准，在全省率先实施了农村居民最低生活保障制度，有效保障了城乡困难群众基本生活。

五年来，我们不断加强政府自身建设，推进民主法制建设，经济社会发展的政治和法制保障更加有力。按照市委统一部署，深入开展保持共产党员先进性教育活动和科学发展观学习教育活动，政府机关干部队伍素质不断提高。修订完善了市政府议事规则和办事程序，进一步健全了公众参与、专家咨询、政府决策相结合的民主决策机制;建立了重大事项社会公示、听证、质询制度，将每年的12月份定为“市民献计献策月”，促进了决策的科学化、民主化。“五五”普法深入开展，全民法制观念进一步增强。认真贯彻落实《行政许可法》和《公务员法》，加强政府法制建设，推进依法行政，强化法制监督，取消行政许可和行政审批项目601项。加强政风和廉政建设，扎实推进行政权力公开透明运行和行风评议，深入实施政府机关“双提”工程，行政效能不断提高。坚持并完善了向人大报告工作、向政协通报情况制度，自觉接受人民代表大会及其常务委员会的依法监督，重视发挥人民政协的政治协商、民主监督和参政议政作用。五年来，承办市以上人大代表批评意见、建议和政协提案3390件，办结率100%，促进了政府工作水平的提高。加强人民群众来信来访工作，广泛推行信访代理制，健全完善矛盾纠纷排查调解工作体系，切实维护了人民群众的合法权益。深入开展“诚信平安唐山”创建活动，强化社会治安综合治理，依法打击各类犯罪活动，坚持不懈狠抓安全生产，维护了全市政治安定和社会和谐稳定。

特别需要指出的是， 2007年是我市贯彻胡锦涛总书记视察唐山重要指示、建设科学发展示范区的起步之年，是落实市第八次党代会和市委八届三次全会精神、开启科学发展新征程的重要一年。一年来，我们坚持以科学发展示范区建设为总揽，以“抢抓新机遇、建设新唐山、实现新跨越”为主题，扎实推进科学发展示范区建设，开创了唐山科学发展的新局面。围绕建设科学发展示范区，广大干部群众思想观念出现了新变化，资源型城市转型取得了新进展，以曹妃甸为重点的沿海开发步入新阶段，对外开放迈出新步伐，新农村建设取得新成效，城市建设改造跃上新台阶，人民群众幸福指数有了新提高。2007年，全市地区生产总值增长15%，全部财政收入增长25.2%，其中地方一般预算收入增长27.7%，全社会固定资产投资增长35.4%，城镇居民可支配收入和农民人均纯收入分别增长15%和13%。全市上下政通人和、心齐气顺，经济发展、社会和谐，各项事业呈现出生机勃勃的崭新局面。

各位代表，回顾五年来的政府工作，面对不断变化的形势，面对新的任务和要求，我们始终坚持解放思想，实事求是，与时俱进，把贯彻落实中央和省工作部署与唐山实际紧密结合，大胆探索，勇于创新，不断丰富完善新唐山发展思路，创造性开展工作;始终坚持把科学发展观作为经济社会发展的根本指针，以科学发展观统领各项工作、指导改革与发展，在全市形成了科学发展的高度共识，形成了推进科学发展的强大合力;始终坚持以经济建设为中心，抢抓机遇，奋勇争先，努力推动经济社会更好更快发展，切实当好建设沿海强省“领头羊”;始终坚持求真务实的作风，坚韧不拔，锲而不舍，以干大事、创大业的气魄和韧劲，狠抓曹妃甸开发建设、资源型城市转型、改革开放等事关唐山科学发展全局的重大问题，带动和活跃了工作全局;始终坚持执政为民，一切依靠人民，一切为了人民，高度关注和改善民本民生，努力促进社会和谐，使改革发展成果惠及全市人民，赢得了广大群众的信赖和支持。

各位代表，过去五年是我市经济社会发展最好的时期之一，为我们今后加速推进唐山科学发展、实现新的跨越奠定了坚实基础。这是党中央国务院、省委省政府和市委正确领导的结果，是全市人民同心同德、锐意进取、艰苦奋斗的结果。在此，我代表市政府，向全市工人、农民、知识分子、各级干部和广大建设者，向人大代表、政协委员，向各民主党派、工商联、无党派人士和人民团体，向驻唐部队、武警官兵、公安政法干警以及关心支持唐山发展的各界朋友，表示崇高的敬意和衷心的感谢!

在充分肯定成绩的同时，我们也清醒地认识到，政府工作中还存在着许多问题和不足，前进道路上还面临着一些突出矛盾和困难。主要是：经济结构性矛盾仍比较突出，服务业发展相对滞后，经济增长的资源环境代价过大，产业集中度、对外开放度不高;支撑科学发展的体制机制还不够健全，人才支撑能力不足，深化改革的任务还十分艰巨;城乡居民总体收入水平还不高，劳动就业、社会保障、物价、安全生产等关系群众切身利益的问题有待进一步解决，城乡面貌、交通、人居、生态环境等诸多方面还不尽如人意;政府自身建设和管理需要进一步加强，政府工作与新形势、新任务要求和人民群众的期望还有不小的差距，一些政府工作人员服务意识不强、办事效率低下，形式主义、官僚主义还比较突出，以权谋私甚至贪污腐败问题仍然存在。对这些问题，我们一定引起高度重视，在今后工作中认真加以解决。

二、今后五年的工作思路和奋斗目标

当前，唐山正处在经济社会加速转型、科学发展示范区建设加速推进的关键时期。纵观时代大势，世情、国情、省情、市情正在发生广泛而深刻的变化。在新的形势下加快唐山跨越发展，机遇前所未有，挑战前所未有，机遇大于挑战。基于对十七大精神的学习领会，基于科学发展示范区建设的实践探索，基于对新形势、新任务的分析把握，基于人民群众的呼声愿望，今年初召开的市委八届四次全会在坚持市委八届三次全会以来形成的一系列发展思路和重要举措的基础上，鲜明提出“开放创新、富民强市，把新唐山建成科学发展示范区、建成人民群众幸福之都”的总战略、总目标。全市上下一定要把智慧和力量凝聚到市委这一重大战略部署上来，进一步增强忧患意识、竞争意识、责任意识，把握大势，抢抓机遇，加压奋进，努力把唐山改革发展推向一个新境界。在未来发展中，我们要认真学习贯彻党的十七大、省委七届三次全会和市委八届四次全会精神，高举中国特色社会主义伟大旗帜，坚持以邓小平理论和“三个代表”重要思想为指导，深入贯彻落实科学发展观，以“抢抓新机遇、建设新唐山、实现新跨越”为主题，以科学发展示范区建设为总揽，以开放创新、富民强市为总战略，坚持好字优先、好中求快，坚持改革开放，坚持以人为本，扎实推进经济、政治、文化、社会建设，为把新唐山建成科学发展示范区、建成人民群众幸福之都而努力奋斗!

开放创新、富民强市，把我市建成科学发展示范区、建成人民群众的幸福之都，是一个长期战略和奋斗目标，必须着眼长远，狠抓当前，分阶段、有步骤地加以推进。今后五年，我们将在全面完成“十一五”规划的基础上，努力实现以下目标：

经济繁荣。到2010年，力争人均国内生产总值比2000年翻两番。资源型城市转型取得重大进展，发展方式实现重大转变，综合竞争力和可持续发展能力显著增强，经济发展质量走在全国前列，建成现代化沿海城市。

社会和谐。安定团结、民主公正、管理有序、诚信友爱、充满活力的和谐社会体系基本建成，全社会法制观念进一步增强，城乡等值化服务体系逐步建立，覆盖城乡居民的社会保障体系基本形成，人人享有基本生活保障。覆盖全社会的公共文化服务体系基本建立，社会主义核心价值体系深入人心，良好思想道德风尚得到进一步弘扬。

生态优美。基本建成资源节约型、环境友好型社会，循环经济形成较大规模，可再生能源利用比重显著上升，实现水、土地和能源等主要资源的低消耗、“三废”排放总量的负增长，生态环境质量明显改善。“十一五”期间，单位生产总值能耗降低20%左右，主要污染物排放总量减少20%。

体制健全。支撑科学发展的体制机制基本形成，市场经济体制更加完善，政府基本公共服务能力显著增强，社会管理体系更加健全。

人民幸福。人民群众幸福指数大幅提升，全民受教育程度和创新型人才培养水平明显提高，社会就业更加充分，合理有序的收入分配格局基本形成，覆盖城乡的多层次、多样化、高水平的医疗服务体系更加健全，人人享有基本医疗卫生服务。

上述发展思路和奋斗目标，是按照市委八届四次全会的战略部署，在全面分析形势、认真研究市情市力基础上确定的，与我市“十一五”规划和全面建设小康社会目标相衔接，通过全市人民共同努力一定能够实现。实现上述目标，全市经济社会发展将跨上一个新台阶，科学发展示范区建设将迈出实质性步伐，人民群众将过上更加美好的新生活，新唐山将焕发出更加蓬勃的生机!

做好今后五年政府工作，必须坚持“六个牢牢把握”：一是必须牢牢把握抢抓新机遇、建设新唐山，在科学发展道路上实现新跨越这一主题。抓住千载难逢的发展机遇，进一步增强加快科学发展示范区建设的责任感、紧迫感，乘势而上，顺势而为，大胆探索，勇于实践，推动我市科学发展再上新台阶。二是必须牢牢把握建设科学发展示范区这个总揽。围绕把我市建成科学发展示范区，广泛开展科学发展示范单位创建活动，扎实推进科学发展示范县(市、区)、示范乡镇、示范园区、示范企业、示范机关建设，把科学发展观要求贯穿于新唐山建设的各个环节、各个方面，在全市兴起科学发展的新热潮。三是必须牢牢把握加快转变经济发展方式这个重点。把转变经济发展方式作为资源型城市转型的核心战略和重中之重，着力破解科学发展难题，加快推进由内陆资源型经济向沿海开放型经济转变，由三次产业各自发展向三次产业融合发展转变，由单一产业支撑向多门类产业拉动转变，由总量扩张型向创新驱动型转变，由资源粗放利用向循环经济转变，由产业节点向产业链条转变，由产业分散发展向产业集群发展转变。四是必须牢牢把握“好字优先、好中求快”这个本质要求。“好”是科学发展最鲜明的特征，好中求快是科学发展的题中之义。要好字优先，努力做到速度与质量效益相统一、发展与资源环境相适应、改革与发展稳定相协调。要好中求快，积极有作为地贯彻中央宏观调控政策，在优化结构、提高效益、降低消耗、保护环境前提下，力求发展的步伐更大、速度更快。五是必须牢牢把握以人为本、改善民生这一根本目的。坚持一切为了人民群众的幸福，在经济快速发展、财政收入不断增加的情况下，更加重视发展成果的普惠性，拿出更多的财力物力，加强以改善民生为重点的社会建设，坚持不懈推进安居、扩大就业、社保扩面、教育扶助、全民健康、文化繁荣、蓝天碧水、政务创优、诚信平安创建、民主参与等“十大幸福工程”，使人民群众共享改革发展成果，得到更多实惠。六是必须牢牢把握提高科学发展能力这一关键。全面提高各级领导班子和领导干部的科学决策能力、科学规划能力、科学管理能力和科学探索能力，使各级干部确有真才实学，确实真抓实干，更好地担负起建设科学发展示范区的重任。

各位代表，开放创新、富民强市，把新唐山建成科学发展示范区、建成人民群众的幸福之都，是全市725万人民的共同愿望。新唐山科学发展的宏伟蓝图已经绘就。只要我们同心同德，齐心协力，求真务实，奋力拼搏，就一定能够谱写出更加辉煌的新篇章!

三、2008年主要工作任务

今年是全面贯彻落实党的十七大精神的第一年，是实施“十一五”规划承上启下的关键年，是全面推进科学发展示范区建设的提速年、攻坚年，做好今年的工作具有特别重要的意义。全市国民经济和社会发展的主要预期目标是：在优化结构、提高效益、降低消耗、保护环境的基础上，地区生产总值增长14%以上;全社会固定资产投资增长25%;全部财政收入增长14%以上，其中一般预算收入增长12.5%;城镇居民人均可支配收入和农民人均纯收入分别增长12%和10%;城镇登记失业率控制在4.5%以下。

按照市委八届四次全会精神，围绕加快科学发展示范区建设，全力推进十项重点工作：

(一)全力推进沿海“四点一带”开发建设。遵循区域经济一体化发展规律，打破行政区划束缚，以港口为依托，以园区建设为载体，以发展临港产业为特色，实施“四点一带”战略，统筹沿海一线开发建设。这是事关我市未来发展最为重大的战略布局。“四点”，即曹妃甸新区、乐亭新区、丰南沿海工业区、芦汉经济技术开发区，“一带”，即贯穿“四点”而形成的沿海经济隆起带。曹妃甸新区建成能源和矿石等大宗货物的集疏港、中国北方新型工业化基地、商业性能源储备基地、国家级循环经济示范区、中国北方商务休闲之都和生态宜居的滨海新城，重点发展现代物流、钢铁、石化、装备制造和海洋化工产业，同时带动高新技术产业和高端服务业快速发展。乐亭新区建成精品钢材生产基地、煤化工产业基地、临港装备制造产业基地、滨海旅游胜地和市区产业转移承载区，重点发展港口物流、精品钢铁、煤化工、装备制造、生态旅游等产业。丰南沿海工业区建成装备制造业基地、新型建材基地、市区产业转移承载区、曹妃甸新区与天津滨海新区的产业对接与配套基地，重点发展装备制造产业、新型建材产业。芦汉经济技术开发区建成唐津两市区域合作的“桥头堡”，曹妃甸新区和天津滨海新区辐射的承接区，重点发展高新技术与信息服务外包产业、自行车配件与五金制品产业。“四点一带”开发建设，要坚持统一领导、统一规划、统一政策、统一资源配置、统一公共服务平台、统一基础设施建设，加速产业聚集，使之成为科学发展先行区、先进产业聚集区、双向开放承载区、改革创新引领区、生态文明样板区、跨越发展支撑区。年内“四点一带”区域规划启动实施，完成投入655亿元。力争用5-10年的时间，使“四点一带”创造的GDP占到全市经济总量的一半，再造一个新唐山。其他县(市)区要与“四点一带”开发建设搞好对接，努力形成海陆互动、优势互补、互利共赢、竞相发展的生动局面。

曹妃甸新区是“四点一带”的核心和龙头。把今年作为曹妃甸新区开发建设的“配套年”，掀起开发建设新高潮。全年完成投资400亿元。加快规划编制配套。编制完善曹妃甸新区产业发展总体规划、曹妃甸生态城建设各项规划和海洋功能区域规划，加紧制定出台曹妃甸新区产业准入标准，为推进曹妃甸新区科学发展提供依据。强力推进基础设施建设配套。抓好曹妃甸工业区综合服务区建设，力争到年底基本具备生活办公条件;全面启动曹妃甸生态城起步区建设。加快工业区路水电讯等基础设施配套，满足开发建设需要。在抓好矿石、原油、煤炭、液化天然气四大专业码头建设、运营的基础上，确保一号港池基本建成投入使用，二、三号港池全面开工建设;加快造地进度，全年新增造地40平方公里。加速推进产业配套。以国家批准曹妃甸循环经济产业发展总体规划为契机，围绕现代港口物流、精品钢铁、化工、装备制造四大主导产业和相关配套产业，加快谋划建设一批高科技含量的重大产业项目，努力建设高新技术支撑下的新型重化工业基地。确保首钢京唐钢铁厂等在建项目如期竣工投产，久泰能源煤化工等一批重大产业项目开工建设。积极推进原油储备基地和大型炼化一体化等项目前期工作。大力推进改革配套。重点围绕实现体制机制配套、金融支撑配套和干部队伍配套，进一步健全完善曹妃甸新区管理体制，全面推进行政管理、投融资体制和开发建设模式以及人才支撑体系等方面综合配套改革。积极争取设立产业发展基金，引进战略投资者，组建曹妃甸控股公司。启动保税仓库和出口监管仓库建设，逐步建立保税物流中心、出口加工区。

(二)全力推进产业结构调整。坚持走新型工业化道路，围绕资源型城市转型，以打造主导产业链为重点，以园区建设为平台，以科技创新为动力，强化政策引导，加速推进产业结构向高端、精品、专业化、深加工方向发展，加快构筑现代产业体系。做大做强主导产业链。力争今年产业链经济增加值占全市经济总量的50%。遵循产业链经济发展规划，全力抓好精品钢材、装备制造、化工、现代农业、现代服务业、高新技术和环保七大主导产业链，其中精品钢材、装备制造、服务业三大产业链务求取得突破。钢铁产业：继续强力推进整合重组和淘汰落后，鼓励支持唐钢、首钢、国丰、津西等优势企业整合地方钢铁企业，做大产业集群，延伸钢铁深加工。全市精品钢比重达到50%。装备制造业：以时速300公里动车组、重型装载机械、成套建材装备制造企业为龙头，以专业化协作配套为途径，实施技术扩散、产品扩散，打造产业链模式。积极寻求战略合作，力促国内外知名装备制造企业落户我市，加快打造中国北方重型装备制造业基地。服务业：抓住国家和省加大对服务业支持力度、北京举办奥运会等有利时机，把振兴服务业作为优化经济结构、转变发展方式的关键环节，制定完善发展规划，出台支持政策，促其加快发展，实现扩容提质。以市中心区“退二进三”为契机，充分利用“金角银边”等黄金地段，积极引进战略合作者。坚持重点发展生产性服务业、创新发展消费性服务业、大力发展新型高端服务业、协调发展城乡服务业的思路，加快发展现代物流、旅游、商务休闲、金融服务、商贸服务、文化创意、软件和信息服务等现代服务业，确保实现新的突破。全年实施重点服务业项目60个，年度计划投资240亿元;服务业增加值增长15%以上。化工、现代农业、高新技术和环保等产业都要按照链状产业体系要求，培育壮大产业链龙头企业，加速产业融合，实现做大做强。壮大园区经济规模。按照“规划科学、定位准确、布局合理、功能健全”的要求，加快园区建设步伐，重点抓好高新技术产业园区等10大园区和唐山市现代装备制造工业区等10大钢铁深加工园区建设，努力提升产业集中度和竞争力。从土地供应、机制创新、设立基金、税收分享等方面研究制定园区经济支持政策，为园区建设创造良好环境。全市园区经济创造的增加值达到1000亿元以上，占全市GDP的比重达到30%左右。加快推进科技创新。围绕提升产业核心竞争力，加快建立以企业为主体、产学研融合互动、技术中介服务发达的科技创新体系，推进创新型城市建设。积极培育建设80家市级以上企业工程技术研发中心、行业重点实验室等科技研发机构，提高自主创新能力。深入推进科技合作，整合科技资源，突破关键共性技术，支持以信息产业为代表的高科技产业快速发展，重点抓好500项关键技术创新和成果转化项目以及150项重大专利技术转化，实施21项重大科技创新工程。深化科技管理体制改革，进一步健全完善支撑科学发展的科技创新机制。

(三)全力推进重点项目建设。坚持把项目建设作为推进资源型城市转型、加快科学发展示范区建设的重要载体，认真贯彻落实国家宏观调控政策，创新发展理念，优化投资结构，保持固定资产投资合理增长。适应宏观调控政策取向，正确引导资金投向，严格控制高耗能、高排放项目，增加对优势产业、新兴产业等结构优化升级项目和经济社会发展薄弱环节、社会事业、改善民生等方面的投资。今年全市计划安排重点建设项目100项，总投资2300亿元，年度计划投资475亿元，增长28%以上。加快冀东油田勘探开发等40个续建项目建设进度，力促日本住友建筑工程机械等50个项目开工建设，确保20个项目年内竣工;抓好120万吨甲醇等10个重大项目的前期工作，争取早日开工。同时，积极谋划一批国家鼓励支持、有利于资源型城市转型、产业链条延伸、带动能力强的重大项目，下大力抓好有利于促进社会就业的劳动密集型中小项目和服务业项目，不断充实项目库。认真落实项目责任制，坚持依法依规上项目。积极破解项目用地、融资等瓶颈制约。通过修订完善建设用地规划、土地置换、生态恢复、整合土地资源等多种方式，探索存量土地挖潜的有效办法，多渠道增加建设用地有效供给;充分考虑国家货币政策趋紧带来的影响，拓宽投融资渠道，深化投融资体制改革，创新投资运营模式，提高资金使用效益。

(四)全力推进节能减排。今年是实现“十一五”节能减排目标的关键一年。坚持把节能减排作为建设科学发展示范区的基础工程，作为建设人民群众幸福之都的关键举措，全力打好节能减排攻坚战、持久战，务求取得明显成效。全市单位生产总值能耗降低4.6%，二氧化硫、化学需氧量分别削减5.5%和6.5%。严格落实节能减排工作责任制。完善能源消耗和主要污染物总量减排统计、监测和考核办法，对未能完成节能减排任务的县(市)区政府实行问责制、试行一票否决制。对纳入省“双三十”和我市“10100”工程的县(市)区和高耗能、高排放企业实施重点监管、考核，确保完成节能减排承诺目标。坚决淘汰落后产能。深入开展节能减排持续攻坚联合行动，进一步加大对钢铁、水泥等高污染、高耗能行业落后产能的淘汰力度，建立健全落后装备、高耗能产业退出机制和政府支持节能减排鼓励机制，确保淘汰落后责任制和对无证非法、高污染、高耗能企业治理整顿责任制的落实。大力发展循环经济。按照“减量化、再利用、资源化”原则，积极开发和推广节约、替代、循环利用和治理污染的先进适用技术，抓紧对现有企业实行循环经济改造，提升企业技术工艺水平。严格新上项目的循环经济准入，通过推进清洁生产、三废利用、产品延伸加工以及跨产业配套利用资源等多种途径，促进资源深度利用，加快建设循环经济产业体系。重点实施以钢铁高炉转炉煤气、水泥窑余热利用、可再生能源为代表的100个节能改造和循环利用项目。加快实施天然气进市区工程，推广新型能源，优化能源结构。切实加强生态环境建设。实施北部山区、城区采煤沉降区、境内水体和沿海湿地四大生态修复工程，深入开展全民植树造林。抓好227个大气环境综合整治和重点行业污染源治理项目，两年内所有县城都必须建成使用污水处理厂。加强土地、水、森林、矿产等资源的保护和节约利用，严厉查处乱采滥挖矿产资源等违法违规行为。增强全社会生态文明观念，动员全市人民更加积极投身于资源节约型、环境友好型社会建设，让唐山的山更绿、水更清、天更蓝。

(五)全力推进新农村建设和城乡一体化进程。以农民增收、农业增效、农村增色为重点，统筹城乡协调发展，加快新农村建设，深入推进城乡一体化进程。多渠道增加农民收入。完善落实各项支农惠农政策。加大对农业农村投入力度，加强农业基础设施建设。推进农业结构调整，大力发展现代农业。加快发展农业产业链经济，培育壮大农业龙头企业，推进农产品精深加工，完善农业服务体系，提高产业化经营水平，农业产业化经营率达到61%。加强重大动植物疫病防控和农业科技推广体系建设，大力发展无公害农产品、绿色和有机食品生产。高度重视农资价格上涨问题，加强农资市场监管。加快发展农村二三产业，积极引导农村劳动力有序转移就业，努力增加农民务工收入。优化农民进城务工环境，加强非农就业培训和服务，实施农村劳动力转移培训“阳光工程”。全年免费培训农民9.8万人，转移农村富余劳动力15万人次。下大力改善农村人居环境。强力推进农村饮水安全工程建设，年内投资5亿元，基本解决农村饮水安全问题;加大农村公路建设力度，改造农村公路780公里;加快构建城乡一体的信息服务网络，建成一批农业特色网站;推进村民中心建设，力争年内全市每个行政村都建有一个村民中心;抓好450个文明生态村创建工作，使开展创建的村达到60%以上;推广普及沼气、太阳能路灯、吊炕、秸秆气化等新型能源和生态循环农业建设，让农村“富起来、暖起来、亮起来、乐起来、循环起来”。全面深化农村改革。稳定和完善土地承包关系，健全土地承包经营权流转市场，发展多种形式的适度规模经营。健全农村金融服务体系，深化农村信用社体制改革，抓好村镇银行和农村资金互助试点，发展农村政策保险和农业商业保险。培育壮大农村专业合作经济组织，鼓励发展农村综合服务组织。深化林权制度改革。加快城乡一体化步伐。打破城乡二元结构，优化城乡产业布局，发展壮大县域经济，加快建立以工促农、以城带乡长效机制，推进城乡规划一体化、建设一体化、产业一体化、公共服务一体化、社会管理一体化。今年全面落实“五个无障碍”：农民工进城落户无障碍，凡是在唐山有合法固定住所、职业稳定的农民工，其配偶、未成年子女均可在唐山落户;农民进城就业无障碍，认真落实公共就业服务政策、就业扶持政策和城乡一体的就业援助政策，建立统一规范的人力资源市场，畅通农民进城务工渠道;农民工子女就读无障碍，只要在我市城镇取得暂住证的农民工，其子女可以就近入学，享受与城镇居民子女同等待遇;农民进城公共交通无障碍，完善路网建设，增加客运班次，延伸通达深度;农民进城就医报销无障碍，健全完善覆盖城乡的新型农村合作医疗信息系统，参合农民到城市看病实行“出院即报”。

(六)全力推进改革创新。推进科学发展示范区建设，关键在体制机制创新。进一步激发政府管理活力。加快行政管理体制改革，健全政府职责体系，形成权责一致、分工合理、决策科学、执行顺畅、监督有力的行政管理体制。探索以职能有机统一的“大部门”体制解决政出多门问题，以制定市权事项审批程序规定解决层次环节多的问题，以落实限时办结制解决效率低下的问题，努力建设服务型政府。进一步清理审批事项，凡适合下放的财权、事权和审批权一律下放，凡不涉及全市总体发展战略的事项一律由县(市)区审批，凡能够通过市场机制解决的问题政府一律不干预。全面推进乡镇机构改革，稳步推进事业单位改革。进一步深化财政改革，加强税收征管，努力增收节支，增强公共服务能力。进一步激发市场主体活力。继续深化国企改革，加快推进57家国有企业改制攻坚，年内完成47家，确保两年内全部完成。改变公用事业单位国有独资经营模式，融合各类资本，逐步实现股权多元化。适时组建唐山投资控股集团公司，提高国有资本运营效率，发挥国有资本的引领、调控、示范和带动作用。以建立健全金融、地产、产权、人力资源等要素市场为重点，进一步完善要素市场体系。创造宽松环境，鼓励全民创业。大力发展民营经济、混合所有制经济，重视支持中小企业发展，在全社会倡导“劳动为本、创业立身”思想观念，引导广大群众主动闯市场、谋发展，把群众中蕴含的追求富裕、勇于创造的潜能释放出来，把社会积淀的民间资本、各种资源有效利用起来，形成活力迸发、全民创业的生动局面。深入实施人才强市战略，创新人才政策机制，加大人才培养引进使用工作力度，最大限度地激发各类人才的创业热情和创造活力。进一步激发金融活力。面对国家银根收紧的新形势，把构建区域性现代金融体系作为建设科学发展示范区的重要战略。继续搞好银企对接，充分发挥国有商业银行主渠道作用。积极引进光大、民生等股份制银行，争取外资银行来我市设立分支机构。加快发展完善资本市场，扩大直接融资，鼓励支持企业上市和发行公司债券，年内力争冀东物贸等企业成功上市。积极发展新型金融组织和金融业务，引导民间融资。加快城市商业银行改革。切实改善金融生态环境，保障金融机构和企业合法权益。

(七)全力推进双向开放。树立强烈的大开放意识，形成以开放促改革、促发展的正确导向，优化开放环境，创新开放平台，在全市上下形成全民招商、参与开放、服务开放的浓厚氛围，构建全方位、多层次、宽领域的大开放格局。加大招商引资力度。坚持非禁即入，鼓励和吸引国内外资本进入我市农业、工业、服务业各个领域;坚持把引进战略投资者作为主攻方向，以沿海大规模开发建设为契机，积极对接世界500强和国内大公司、大集团，重点谋划推介30个超千万美元利用外资大项目，力争取得实质性进展;坚持引资金、引技术、引智力与引进先进管理经验并重，不断拓展双向开放的广度和深度;坚持注重发挥企业的主体作用，真正把企业推向对外开放的主战场。全年实际利用外资8亿美元，增长20%。扩大对外贸易。加快转变外贸增长方式，优化出口产品结构，推动机电、陶瓷等优势产品出口由数量规模型向质量效益型转变。全市进出口总额增长20%以上，其中出口增长15%以上。加强区域合作。以更加积极主动的姿态，深入推进与环渤海地区尤其是与京津的全方位合作，积极承接产业转移，继续拓展与中西部地区的合作，实现优势互补，互利共赢，共同发展。优化开放环境。进一步增强“亲商、安商、扶商”意识，用优良的发展条件吸引投资者、用优质的服务留住投资者、用完善的法制保护投资者。全力办好首届科学发展(曹妃甸)论坛，高水平筹备组织第十一届唐山中国陶瓷博览会，扩大唐山知名度，提高唐山开放度。

(八)全力推进新型城镇化建设。着眼于全市经济社会发展全局，着眼于建设适宜人居和创业的幸福家园，拓展城市空间，完善城市功能，突出城市特色，提高城市品位，构筑新型城镇化发展格局。在巩固去年城市建设改造攻坚战成果的基础上，进一步加大城市规划、建设和管理力度，以建设曹妃甸生态城、凤凰新城、南湖生态城和空港城四大主体功能区为重点，全面实施“14421”工程，努力打造魅力唐山、宜居唐山。全年城建总投资264亿元，其中市中心区162亿元。抓好“一号工程”。加快震后危旧平房改造和经济适用住房、廉租房建设，努力满足老百姓的住房需求。抓好“四大主体功能区”建设。曹妃甸生态城功能区，以建设世界一流的生态城市、港口城市、滨海城市、示范性城市、国际性城市和环渤海中心城市为目标，积极推进与意大利、德国、韩国等国家的合作，抓紧启动城市基础设施建设，争取3-5年形成基础框架。凤凰新城功能区，以建设现代商务中心、总部中心为目标，抓紧启动总部基地一期、规划展馆、青少年宫等项目，规划建设一批体量大、造型新、功能全的标志性建筑，尽快形成大规模开发建设热潮。南湖生态城功能区，以建设在国内享有较高知名度和美誉度的休闲度假胜地、文化创意园区为目标，制定整体规划和各专项规划，启动拓展湖面、环城水系建设工程，加快推进地震遗址公园、国家矿山公园等重点项目建设，大力开展植树造林、景观绿化，努力打造独具特色的城市品牌。空港城功能区，以建设北方航空货物分拨配送中心为目标，抓紧编制《空港城总体规划》，统筹发展空港物流业和高新技术产业，开辟唐山通向世界的空中通道。抓好城市“四化”。围绕提高城市绿化、亮化、美化、净化水平，深入推进“三山三河两湖”环境综合改造，实施建设路、新华道等城市主干道沿线建筑改造和街景美化亮化，深入开展环境卫生综合整治。抓好“两项改造工程”。启动市区企业搬迁改造工程，制定实施市中心区企业搬迁改造规划，逐步将高耗能、高污染企业向优势区域转移;加快推进市区城中村改造工程，改善群众居住条件，整体提升城市档次。抓好机场通航。抓紧唐山机场军民两用跑办工作，力争尽早实现民航正式通航。同时，加快城乡路网建设。实施北新道综合改造，新建延伸朝阳道等15条道路、翻修银河路等9条道路;加快城乡干线公路建设，确保城市外环线、唐曹高速、沿海高速连接线全面建成通车，唐丰快速路等8条公路全面开工，谋划建设沿海公路等6条公路，实施农村公路“123”工程，构建快速便捷的城乡现代化交通网络。积极推进县城和小城镇建设。支持迁安、遵化等县(市)向中等城市迈进，其它各县城进一步扩大规模，完善功能，加强管理，提升档次，加快培育一批人口在5万以上的小城镇，构建独具唐山特色的城镇体系。

实施“14421”工程，科学规划是龙头，创新建设是关键，加强城市管理是保证。坚持以超前的理念抓规划。引进国际一流的设计机构，进一步完善城市新一轮总体规划、重点区域详规等规划，充分体现城市规划的前瞻性、先导性和科学性。牢固树立“规划即法”理念，切实维护规划的权威性和严肃性。坚持以创新的精神抓建设。全面放开城市建设市场，探索建立多元融资机制、全方位经营城市机制、土地市场一级开发机制，大力引进国内外知名的战略投资者参与城市建设，推行财政投资建设项目代建制。坚持以改革的办法抓管理。科学界定市、区两级管理权限，将城市管理重心下移到区和街道办事处。实行综合执法和数字网格化城市管理，进一步提高城市管理规范化、信息化水平。

(九)全力推进各项社会事业发展。在发展经济的同时，更加重视社会建设，不断加大社会事业投入力度，促进经济社会协调发展。今年全市财政投入社会事业资金增长14.7%。优先发展教育事业。围绕实现教育公平，进一步整合教育资源，优化教育布局，加大教育投入，促进各类教育均衡发展。巩固提高“普九”成果，深化“以县为主”的教育管理体制改革，实施农村和城市中小学远程教育工程，改造农村陈旧校舍10万平方米。优化高中阶段教育资源，普及高中免费教育。适应经济社会发展需求，积极发展高等教育，支持河北理工大学、华北煤炭医学院新校区建设和博士点申报工作，加快唐山师范学院、唐山学院新校区建设，谋划曹妃甸大学园区。坚持以就业和市场需求为导向，大力发展职业教育，积极培养高素质技能型人才。完善成人教育体系，积极发展民办教育。建立教师队伍培训提高长效机制，提升教师队伍素质。推进文化大繁荣、大发展。加强社会主义核心价值体系建设，积极推进和谐文化建设。大力弘扬新时期唐山人文精神，深入开展向宋志永等13位同志先进事迹学习活动，不断赋予新的时代内涵。高度重视和加强青少年思想道德建设。加快发展文化产业，实施“项目带动、文化创新、文艺精品、文化繁荣”重点工程，谋划启动博物馆、工人文化宫、广电大厦、报业大厦、陶瓷创意文化产业博览区等一批重点文化项目建设，组织推出《李大钊》、《百年窑变唐山瓷》、《近代工业摇篮》等一批优秀影视作品和文艺作品，办好第六届评剧艺术节、第七届唐山市社区文化艺术节等大型文化活动。深化文化体制改革，制定落实鼓励文化产业发展的政策措施，形成多渠道、多元化的投入机制。大力发展医疗卫生事业。健全完善以乡镇卫生院为重点的农村三级医疗卫生服务网络，加快城市社区卫生服务体系建设，提高基层医疗服务水平。城市社区卫生服务覆盖率达到100%。抓好市工人医院外科手术楼、市人民医院肿瘤大楼、市二院门急诊手术综合楼等项目建设，谋划建设凤凰新城大型综合医院。加强传染病预防控制，提高突发公共卫生事件应急处置能力。强化卫生执法监督，规范药品流通秩序，确保人民群众就医用药安全。积极发展体育事业。以迎2008北京奥运会为契机，广泛开展全民健身运动，提高竞技体育水平，加快发展体育产业。精心组织好奥运火炬在唐山传递活动。实施计生惠民工程，稳定低生育水平，提高人口素质。加快推进广播电视数字化发展。继续做好国防动员、人民防空工作，加强民兵预备役建设，深入开展双拥共建活动。重视发展民族宗教、外事侨务、气象、地震、档案、老龄、妇女儿童、残疾人等各项事业，促进社会全面进步。

(十)全力推进民生改善。以提高人民群众幸福指数为出发点，围绕落实“十大幸福工程”，继续为群众办一批实事，切实解决群众最关心、最直接、最现实的利益问题，努力使全市人民劳有所得、学有所教、病有所医、老有所养、住有所居、贫有所扶，共享改革发展成果。劳有所得：建立健全企业职工工资正常增长和支付保障机制，适时提高最低工资标准，努力增加城镇职工收入。落实职工带薪休假制度。深入实施扩大就业工程，抓好就业促进法和劳动合同法的贯彻，完善政策、鼓励创业，以创业带动就业。建立健全困难群众就业援助长效机制，重点解决零就业家庭就业，提高就业的稳定性。加强职业技能培训，提高就业本领。做好高校毕业生、退伍转业军人安置就业工作。全年城镇新增就业岗位6万个以上，下岗失业人员再就业2.6万人以上。学有所教：在农村全部实行免费义务教育的基础上，今年在城市全部免除义务教育阶段学杂费，同时全部免除义务教育阶段农村学生和城市低保家庭学生教科书费，进一步完善各级各类学校贫困家庭学生资助机制。病有所医：健全完善新型农村合作医疗制度，提高农民参合比例和报销比例，使参合农民更多受益;加快推进城镇居民医疗保险制度，参保人数达到70% 以上，妥善解决困难企业职工参加医疗保险问题，在全省率先实现“全民医保”。老有所养：进一步完善养老保险制度，再连续三年提高企业退休人员养老金水平。扩大农村养老保险试点范围，在迁安开展试点的基础上，扩大到遵化、迁西、唐海、丰南、开平、高新区6个县(市)区。完善落实失地农民养老保险制度。住有所居：扎实推进危旧平房改造，年内开工151万平方米。加快经济适用住房建设，扩大廉租房保障范围，切实解决低收入家庭住房问题。年内市中心区开工经济适用住房40万平方米，其中廉租房5万平方米。同时，加强房地产市场调控和监管，增加住房有效供给，使商品房价格稳定在合理水平。贫有所扶：全面落实城乡居民最低生活保障制度，进一步提高城乡低保标准。健全社会救助体系，发展社会慈善事业。深入开展“真情暖万家”、“送温暖、献爱心”活动。

社会稳定和谐，是人民群众安居乐业的基础。高度重视并切实维护好公共安全，健全社会治安防控体系，加强社会治安综合治理，强化全民法制教育，依法防范和严厉打击违法犯罪活动。妥善处理新形势下的人民内部矛盾，加强社会矛盾纠纷排查调处，深入推行信访代理制，带着感情做好群众工作。完善应急预案体系，提高预防和处置突发公共安全事件的能力。加强食品安全监管，确保人民群众食品安全。坚持不懈抓好安全生产，坚决遏制重特大安全事故发生，维护人民群众的生命财产安全。

四、切实加强政府自身建设

新一届政府肩负着建设科学发展示范区、建设人民群众幸福之都的光荣使命。我们必须围绕建设服务型政府，以开展科学发展示范机关创建活动为契机，努力提高“五种能力”，树立“爱民、公正、高效、廉洁”形象，全面提高行政管理和服务水平，开创政府各项工作新局面。

解放思想，与时俱进，提高科学发展能力。紧密围绕加快建设科学发展示范区，以学习贯彻党的十七大精神为重点，深入推进思想大解放，以思想的解放引领观念创新、推动科学发展。按照市委统一部署，扎实开展好学习实践科学发展观活动，进一步增强贯彻落实科学发展观的自觉性和坚定性，进一步解决影响和制约科学发展的突出问题，进一步创新科学发展的体制机制，进一步提高组织推动科学发展的能力。抓好公务员队伍建设，强化素质锻炼，不断提高服务人民群众的本领。

转变职能，全面履责，提高公共服务能力。创新行政管理体制，加快推进政府职能转变，全面正确地履行政府职能。在加强和改善经济调节、市场监管的同时，更加注重社会管理和公共服务，维护社会公正和社会秩序，促进基本公共服务均等化，努力为人民群众提供方便、快捷、优质、高效的公共服务。扎实开展“效率年”活动，进一步加强机关效能建设。继续减少和规范行政审批，推进政务创优工程，完善行政服务中心“一站式”服务制度，开展网上审批试点，进一步降低行政成本，努力提高行政效率和公共服务水平。

强化监督，公开透明，提高依法行政能力。自觉接受人大法律监督、政协民主监督和社会监督，完善和加强政府内部监督，确保行政权力依法公正行使。深入推进行政权力公开透明运行，自觉接受群众监督。健全社情民意反映制度和重大事项专家咨询、社会公示、听证和质询制度，切实做到科学决策、民主决策。继续深入开展民主评议行风政风活动。坚持依法行政，严格按照法定权限和程序行使职权、履行职责，自觉运用法律手段调节经济和社会事务。加强行政立法，推动政府各项工作法制化。改革行政执法体制，减少执法层级，完善执法程序，强化执法监督，切实解决多头执法、执法不公甚至执法违法等问题。

求真务实，埋头苦干，提高工作落实能力。牢固树立正确的政绩观，少说多做、力戒浮躁，坚决反对形式主义、官僚主义，多干打基础、利长远的事，努力创造经得起实践和历史检验的政绩。大力弘扬求真务实之风，实干、善干，敢于负责，勇于突破，创造性地推动工作落实。从市政府各部门做起，大力精简会议、文件和事务性活动。进一步建立健全抓落实工作机制，全面落实目标管理责任制，推行政府绩效管理和行政问责制，完善部门之间协调配合机制，以责任明确的硬任务、真督实查的硬措施、奖罚分明的硬机制，形成干事创业的强大合力。

执政为民，拒腐防变，提高廉洁从政能力。时刻把人民群众的冷暖挂在心上，坚持深入基层、深入实际、深入群众，倾听群众呼声，体察群众疾苦，尽心竭力为群众办实事、办好事、解难题，真正做到权为民所用、情为民所系、利为民所谋。坚持标本兼治、综合治理、惩防并举、注重预防的方针，在坚决惩治腐败的同时，更加注重治本、更加注重预防、更加注重制度建设。坚持不懈开展反腐倡廉教育，筑牢拒腐防变的思想道德防线。认真坚持党风廉政建设责任制，进一步健全和落实“一岗双责”制度。强化对行政权力运行的监督和制约，完善政府投资监管、国有资产监管和非经营性资产管理制度，规范建设工程招投标、经营性土地使用权出让、政府采购和产权交易制度，用制度管权、管事、管人。加大专项治理力度，重点解决环境保护、食品药品安全、安全生产、土地征用与房屋拆迁等方面群众反映强烈的问题，坚决纠正损害群众利益的不正之风。坚持从严治政，廉洁从政，努力在人民群众中树立良好形象。

各位代表，回顾过去辉煌成就令人鼓舞，展望未来宏伟蓝图催人奋进。让我们以党的十七大精神为指导，高举中国特色社会主义伟大旗帜，紧密团结在以胡锦涛同志为总书记的党中央周围，在省委、省政府和市委的正确领导下，解放思想，开拓创新，团结一致，扎实苦干，为把新唐山建成科学发展示范区、建成人民群众幸福之都而努力奋斗!
